# Soshiho-tang for treating common cold in children younger than 12 years: A systematic review and meta-analysis of randomized controlled trials

**DOI:** 10.1097/MD.0000000000013045

**Published:** 2018-11-09

**Authors:** Jieun Jung, Jihun Park, Jun-Yong Choi, Ju Ah Lee

**Affiliations:** a College of Korean Medicine, Gachon University, Seongnam; b Korean Medicine Hospital of Pusan National University; c School of Korean Medicine, Pusan National University, Yangsan, Republic of Korea.

**Keywords:** common cold, protocol, Soshiho-tang, systematic review

## Abstract

**Background:**

Soshiho-tang (SST) is widely used to treat common cold in East Asian countries. Many clinical trials assessing the efficacy and safety of SST formulas for the treatment of pediatric common cold have been reported. The aim of this systematic review is to assess the available clinical evidence on the use of SST formulas in the treatment for common cold in children younger than 12 years.

**Methods:**

Fifteen databases will be searched from their inception until March 2019. We will be including the randomized controlled trials (RCTs) assessing all types of SST formulas used in the treatment of common cold in children younger than 12 years. The methodological qualities of the RCTs will be assessed using the Cochrane Collaboration tool for assessing the risk of bias, while confidence in the cumulative evidence will be evaluated using the Grading of Recommendations Assessment, Development and Evaluation instrument.

**Ethics and dissemination:**

This systematic review will be published in a peer-reviewed journal and will also be disseminated electronically and in print. The review will be updated to inform and guide health care practices.

## 1 Introduction

Cough due to acute upper respiratory infections in children can be caused by various viruses and bacteria. More than 90% of these infections are caused by viruses, such as influenza virus, rhinovirus, para-influenza virus, respiratory syncytial virus, and adenovirus. Main bacteria causing pediatric acute upper respiratory infections include hemolytic streptococcus, pneumococcus, and mycoplasma. The bacterial and viral infections can coexist.^[1]^

Children have minimal resistance to cough and fever owing to the low levels of immunity present in them. The common symptoms of pediatric acute upper respiratory infections include cough, fever, nasal obstruction, rhinorrhoea, fatigue, and tonsillar edema.^[2]^

Young children have short nasal cavity with soft mucous membrane and increased vascularity. Their sinuses, pharynx, and larynx are not completely developed, so they are vulnerable to diseases. If a child has poor nutritional status, dyspepsia, or diarrhea, he or she can have vitamin D, iron, and zinc deficiency.^[3]^

If a doctor fails to provide timely and appropriate treatment for pediatric cough, it can result in severe infection, which could be contagious.^[3]^ The treatment for pediatric cough should be effective and with no side effects.^[1]^

The US Food and Drug Administration recommended that commonly used cough medications should be avoided in children younger than 2 years. In addition, cough medicines such as echinacea are known to be ineffective in children. Even low-dose inhaled corticosteroids and oral prednisolone have no beneficial effects in the treatment of cough in non-asthmatic children.^[4]^

In East Asian medicine, the lung and spleen of children are considered as incomplete, undeveloped organs. The symptoms of a pediatric cough can vary and can be easily affected by environmental factors such as variations in atmospheric temperature. East Asian medicine uses acupuncture, moxa treatment, and herbal medicine formulas to treat pediatric cough.

Clinical studies had reported beneficial effects of herbal medicine formulas in the treatment of cough in children.^[5]^ Studies that administered Soshiho-tang (SST) for the treatment of pediatric cough had reported remarkable results. SST can avoid the damage of gastrointestinal tract that can occur with commonly used cough medications and enable faster recovery of the body and organs.^[3,5]^

Most of the clinical research on SST have been focused on the treatment and prevention of liver diseases such as hepatic fibrosis and liver carcinoma. There are only a few studies which addressed the use of SST in treating cough among pediatric patients under 12 years of age. In this study, we will be systematically reviewing the randomized controlled trials (RCTs) assessing the effectiveness and safety of SST in the treatment of cough in children younger than 12 years.

## 2 Methods

This study protocol report adheres to the PRISMA-Protocols (PRISMA-P) 2015 checklist.^[6]^

### 2.1 Ethics

This study is not a clinical study. Therefore, ethical approval is not required as per our institutional ethics board policies.

### 2.2 Eligibility criteria

Types of studies: Prospective randomized controlled trials (RCTs) that evaluate the effectiveness of SST for common colds in children <12 years of age will be included in this review. Both treatment with SST alone and concurrent treatment of SST and another therapy will be considered acceptable if SST is applied to the intervention group only and any other treatment is provided equally to both groups. Trials with any type of control intervention will be included. No language restrictions will be imposed for the selection of studies.

Types of participants: Children under 12 years of age will be eligible for inclusion. Participants who have a common cold associated with complications or other diseases such as pneumonia and asthma will be excluded. Adolescents (age: 12–18 years) and adults (older than 18 years) will be excluded. We will be including participants belonging to both sexes and with all types of symptom severity.

Types of interventions: Studies that evaluate any type of formulas (i.e., decoction, extracts, pills, or powder) of SST will be eligible for inclusion. The compositions of these formulas will be reviewed, and interventions involving herbal combinations that differ from the original SST formula will be excluded from this review.

### 2.3 Information sources

The following databases will be searched from inception to the present date: Medical Literature Analysis and Retrieval System Online (MEDLINE), Excerpta Medica database, the Cochrane Central Register of Controlled Trials, Allied and Complementary Medicine Database, and Cumulative Index to Nursing and Allied Health Literature. We will also search 6 Korean medical databases (OASIS, the Korean Traditional Knowledge Portal, the Korean Studies Information Service System, KoreaMed, the Korean Medical Database and DBPIA) and 3 Chinese databases, including China National Knowledge Infrastructure (the China Academic Journal, the China Doctoral Dissertations and Master's Theses Full-text Database, the China Proceedings of Conference Full-Text Database, and the Century Journal Project), Wanfang, and VIP. In addition, we will search a Japanese database and conduct non-electronic searches of conference proceedings.

### 2.4 Study records

Data management selection process: Hard copies of all articles will be obtained and read by all authors. Two authors (JJ and JP) will perform the data extraction and quality assessment using a predefined data extraction form. In addition, all interventions applying acupuncture will be extracted using the Standards for Reporting Interventions in Clinical Trials of Acupuncture. Disagreements will be resolved by discussion between all authors. When disagreements regarding selection cannot be resolved through discussion, an arbiter (JAL and JC) will make the final decision. We will contact the corresponding authors of studies with missing information to acquire and verify the data, whenever possible.

### 2.5 Data items

#### 2.5.1 Outcomes and prioritization

Primary outcomes:

- Response rate
- Duration of fever
- Cough

Secondary outcomes:

- Adverse events
- Change in symptoms such as, sore throat, rhinitis, rhinorrhoea, cough, and fever
- Information related to SST usage
- Pattern in response based on traditional Korean medicine (TKM) or traditional Chinese medicine (TCM) therapy
- Range of dosage of SST in each study

### 2.6 Assessment of risk of bias in individual studies

We will independently assess the risk of bias in the included studies, according to the criteria in the Cochrane Handbook, version 5.1.0; these criteria include random sequence generation, allocation concealment, blinding of participants and personnel, blinding of outcome assessment, incomplete outcome data, selective reporting, and other sources of bias.^[7]^ The results of the assessments will be presented using scores of “L,” “U,” and “H,” indicating a low risk of bias, an uncertain risk of bias, and a high risk of bias, respectively.

### 2.7 Data synthesis

Differences between the intervention and control groups will be assessed. Mean differences (MDs) with 95% confidence intervals (CIs) will be used to measure the effects of treatment for continuous data. We will convert other forms of data into MDs. For outcome variables on different scales, we will use standard MDs with 95% CIs. For dichotomous data, we will present treatment effects as relative risks (RRs) with 95% CIs; other binary data will be converted into RR values.

All statistical analyses will be conducted using Cochrane Collaboration's software programme Review Manager (RevMan, version 5.3. Copenhagen: The Nordic Cochrane Centre, the Cochrane Collaboration, 2014) for Windows. When appropriate, we will pool the data across studies to conduct a meta-analysis using fixed or random effects. We will use GRADEpro software from Cochrane Systematic Reviews to create a “Summary of Findings” table.

#### 2.7.1 Unit of analysis issues

For crossover trials, data from the first treatment period will be used. For trials that assessed more than one control group, the primary analysis will combine data from each control group. Subgroup analyses of the control groups will be performed. Each patient will be counted only once in the analyses.

#### 2.7.2 Addressing missing data

Intention-to-treat analyses including all randomized patients will be performed. For patients with missing outcome data, last observation carry-forward analysis will be performed. When individual patient data are initially unavailable, we will review the original source or the published trial reports for these data.

#### 2.7.3 Assessment of heterogeneity

Based on the data analysis, we will use random or fixed-effect models to conduct the meta-analysis. Chi-squared and I-square tests will be used to evaluate the heterogeneity of the included studies. *I*^2^ values >50 will indicate a high heterogeneity. When heterogeneity is observed, subgroup analyses will be conducted to explore the possible causes.

#### 2.7.4 Assessment of reporting biases

Funnel plots will be generated to detect the reporting biases when a sufficient number of included studies (at least 10 trials) is available.^[8]^ However, as funnel plot asymmetries are not equivalent to publication biases, we will aim to determine the possible reasons for any asymmetries in the included studies, such as small-study effects, poor methodological quality, and true heterogeneity.^[9]^

### 2.8 Confidence in cumulative evidence

The strength of the body of evidence will be evaluated using the Grading of Recommendations Assessment, Development, and Evaluation instrument.

## 3 Discussion

SST is a widely used herbal medicine in various Asian countries including China, Japan, and Korea.^[10,11]^ SST consists of 7 herbal ingredients: *Bupleurum falcatum* Linne (12 g), *Scutellaria baicalensis* Georgi (8 g), *Panax ginseng* C. A. Mey (4 g), *Pinellia ternata* Breitenbach (4 g), *Glycyrrhiza uralensis* Fisch. or *Glycyrrhiza glabra* L. (2 g), *Zingiber officinale* Roscoe (3 g), and *Zizyphus jujuba* Miller var. *inermis* Rehder (2 g).

SST is used clinically for symptoms such as, cough, nasal congestion, and runny nose. After administering SST, these symptoms are alleviated, and body temperature and sweat are normalized.^[5]^ SST is a herb that has strengthening effect on the spleen and stomach, and has specific inhibitory effects on viruses and bacteria.^[10]^*B falcatum* Linne has anti-inflammatory and anti-cold effects. *S baicalensis* Georgi eliminates the heat. *Z officinale* Roscoe and *P ternata* Breitenbach alleviate the symptoms of cold by warming the middle chest, smoothening the fluids, harmonizing the stomach, and relieving the nausea. *G uralensis* Fisch. harmonize the effects all medicines. In clinical application, increasing and reducing the doses of the constituent medicines according to different types of syndromes can produce good treatment results.

There are various forms of SST available, such as capsules, tablets, decoctions, and extracts. All types of SST formulas will be included in this study. To date, no systematic reviews of the effects of SST formulas on common colds in children under 12 years of age have been published. This systematic review will provide a summary of the current evidence related to the effectiveness of SST formulas in the treatment of the symptoms of common cold in this age group. In this review, we intend to identify the subtypes of common cold for which this remedy is particularly useful (e.g., certain types of cold based on TKM or TCM theory), the range of dosages, and the modifications in formulas used to improve the effectiveness, with respect to the duration of treatment. The original SST formula is composed of 7 herbs as mentioned above; however, the primary studies show a high heterogeneity in the ingredients of SST. Therefore, we will investigate the composition of each formula used in the primary studies. This evidence will provide useful information to practitioners, parents, and health policy makers regarding the use of SST for the treatment of common cold in children under 12 years of age.

## Author contributions

JJ and JP conceived the study, developed the criteria, searched the literature, analyzed the data, and wrote the protocol. JP conducted the preliminary search. JJ assisted in searching the Chinese literature and extracting the data. JAL and JC revised the manuscript. All authors have read and approved the final manuscript.

**Conceptualization:** Ju Ah Lee.

**Funding acquisition:** Jun-Yong Choi.

**Investigation:** Ju Ah Lee.

**Methodology:** Ju Ah Lee.

**Validation:** Jun-Yong Choi.

**Visualization:** Ju Ah Lee.

**Writing – original draft:** Jieun Jung, Jihun Park.

**Writing – review & editing:** Ju Ah Lee, Jun-Yong Choi.
